# All that glitters is not gold: An interpretive framework for diagnostic test evaluation using *Ascaris lumbricoides* as a conceptual example

**DOI:** 10.1371/journal.pntd.0012481

**Published:** 2024-09-26

**Authors:** Matthew Denwood, Søren Saxmose Nielsen, Abbey Olsen, Hayley E. Jones, Luc E. Coffeng, Gustavo Landfried, Martin K. Nielsen, Bruno Levecke, Stig Milan Thamsborg, Paolo Eusebi, Eleftherios Meletis, Polychronis Kostoulas, Sonja Hartnack, Berra Erkosar, Nils Toft

**Affiliations:** 1 Department of Veterinary and Animal Sciences, Faculty of Health and Medical Sciences, University of Copenhagen, Copengagen, Denmark; 2 Population Health Sciences, Bristol Medical School, University of Bristol, Bristol, United Kingdom; 3 Department of Public Health, Erasmus MC, University Medical Center Rotterdam, Rotterdam, the Netherlands; 4 Universidad de Buenos Aires, Facultad de Ciencias Exactas y Naturales, Departamento de Computación, Buenos Aires, Argentina; 5 M.H. Gluck Equine Research Center, Department of Veterinary Science, University of Kentucky, Lexington, Kentucky, United States of America; 6 Department of Translational Physiology, Infectiology and Public Health, Faculty of Veterinary Medicine, Ghent University, Ghent, Belgium; 7 Independent Researcher, Foligno, Italy; 8 Faculty of Public & One Health, University of Thessaly, Karditsa, Greece; 9 Section of Epidemiology, Vetsuisse Faculty, University of Zurich, Zurich, Switzerland; 10 FIND, Geneva, Switzerland; 11 Toft Analytics ApS, Værløse, Denmark; University of Calgary, CANADA

## Background

An important step in controlling neglected tropical diseases (NTDs) is to develop new diagnostic tests for surveillance of populations. However, evaluating new tests using an imperfect reference test as a “gold standard” yields biased estimates of sensitivity and specificity [1]. This is due to a mismatch between the implicit case definition of “reference test outcome” and the target condition of “infection status”. For example, direct detection of ectoparasites in a skin scrape yields a reference test outcome of “parasite detected in the sample”; assuming this test is perfect results in a negative skin scrape from a parasitised individual being incorrectly classified as a true negative, which negatively biases the estimated specificity of comparator tests. If the reference test (or combination of tests) provides a sufficiently accurate representation of the target condition, then the sensitivity and specificity of new diagnostic tests can be determined against a presumed gold standard with minimal bias [1,2]. However, this scenario seems unlikely for many NTDs, and we follow previous authors in recommending that the quest for a perfect reference test be abandoned [3–5].

Latent class models (LCM) provide an alternative method of analysis that is able to use information provided by imperfect tests without introducing bias [6]. These methods can also account for a lack of conditional independence between pairs of tests due to similar or overlapping mechanisms of action that cause the test results to be more correlated than expected [7–10]. LCMs have been used to evaluate diagnostic tests for NTDs [11–13] and are recommended over the use of composite reference tests and panel diagnosis [3].

Importantly, the sensitivity of the current best-performing tests is known to vary between clinical and epidemiological settings for many NTDs, including soil-transmitted helminths (STH; *Ascaris lumbricoides*, *Trichuris trichiura*, *Necator americanus*, and *Ancylostoma duodenale*) [13–17], where the typical intensity of infection varies among the populations of individuals sampled in these settings. It is therefore crucial when evaluating diagnostic tests to consider the purpose of testing and the population in which the diagnostic test will be used when evaluating diagnostic tests [18–20], yet this is often lacking from NTD studies. Further guidance and increased awareness of these issues within the NTD literature is therefore needed.

Here, we present an interpretative framework for LCM, illustrated with a conceptual example of diagnostic testing for *A*. *lumbricoides*. Our goal is to help the reader consider the important biological processes when designing a study and interpreting the estimates obtained. We note that our focus is on individual-level diagnosis and not community-level diagnosis, which is an important distinction in the context of STH.

## Target condition and population

The performance of any diagnostic test should only be interpreted in the context in which it will be used in practice, so we start by defining the purpose of testing. The most relevant target condition for *A*. *lumbricoides* is that of patent infection (the presence of egg-producing females), and for our conceptual example, we include a coproantigen test designed to detect antigen biomarkers from juvenile and adult parasites, 2 egg detection tests (Kato-Katz and mini-FLOTAC), and a PCR test for parasite DNA (from juveniles, adults, and eggs). We assume that all tests are used on separate stool samples from the same individual [21] and that results are binary, i.e., that a cutoff is applied to the egg counts [13]. This is necessary to define sensitivity and specificity, but further work is needed to utilise the count data provided by egg counting methods more effectively [22].

We then identify the target population reflecting where the test would be used in practice, noting that the same test may perform differently across populations due to biological factors such as the intensity of infection and presence of coinfections that may affect sensitivity and specificity, respectively. LCMs typically use data from a minimum of 2 separate groups of individuals, but we recommend a minimum of 3, each with a different expected prevalence. It is important that these reflect the specified target population, which often precludes samples from a known low-prevalence population, as the sensitivity and/or specificity of the tests may vary between endemic and non-endemic settings [15]. We define our target population as school children in endemically infected communities in Ethiopia and subdivide the population by school to generate nonoverlapping groups of children with varying expected prevalence.

## Characterising biological pathways

**Fig 1:**
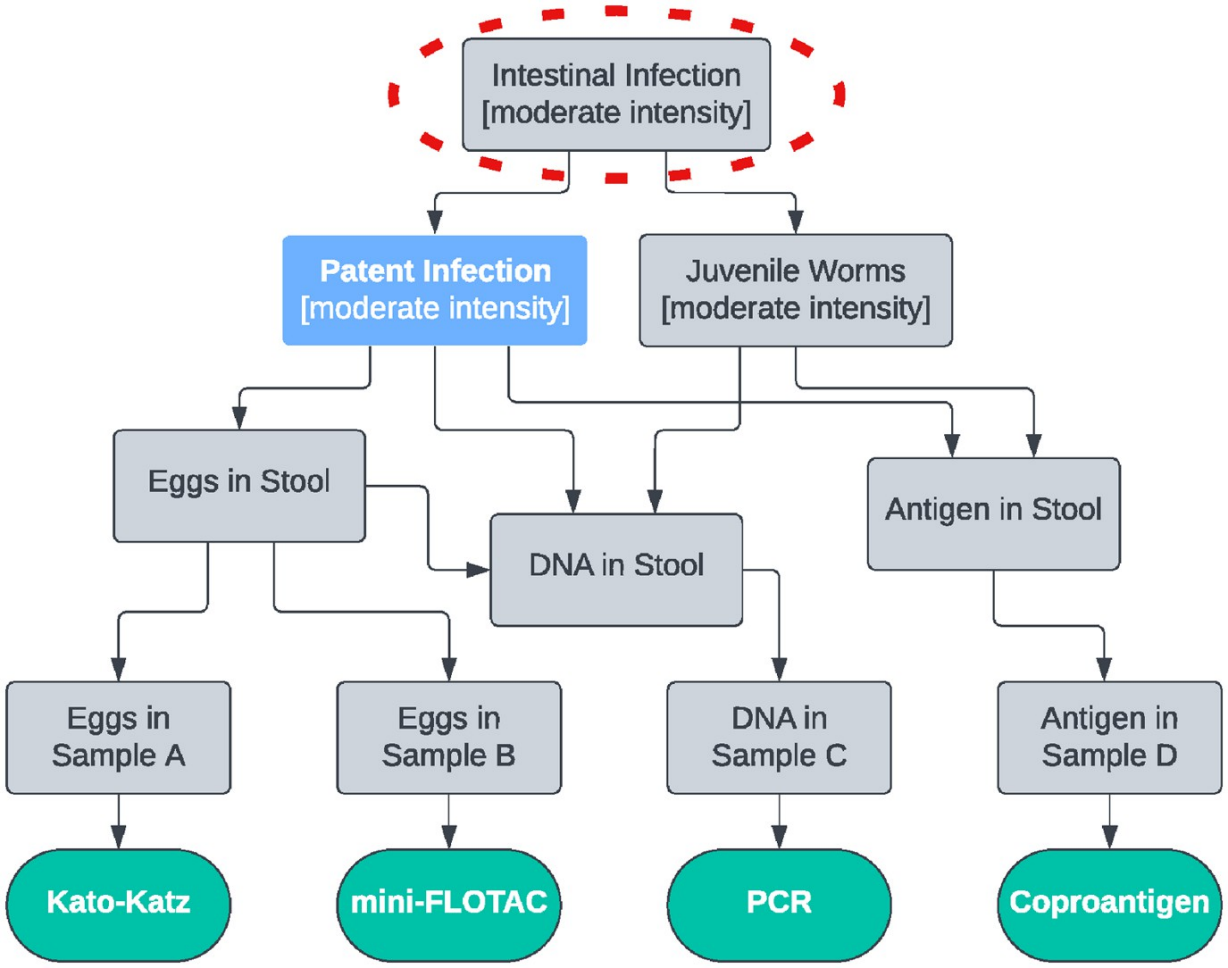
The biological relationship between the stated target condition (blue), unobserved states (grey), and test results (green) for 4 different tests on separate stool samples. The approximate case definition is circled in red.

The life cycle of *A*. *lumbricoides* involves migration of larvae from the large intestine via the lungs and trachea to the small intestine, where juvenile worms develop into adults and produce eggs. A graphical illustration can be used to obtain an overview of the biological link between the target condition, relevant intermediate biological processes, and the observed result of each individual diagnostic test used. We recommend using directed acyclic graphs [DAGs; 23] to represent the relationship between each of these states, with sub-text used to clarify the target population where relevant (Fig 1).

**Fig 2:**
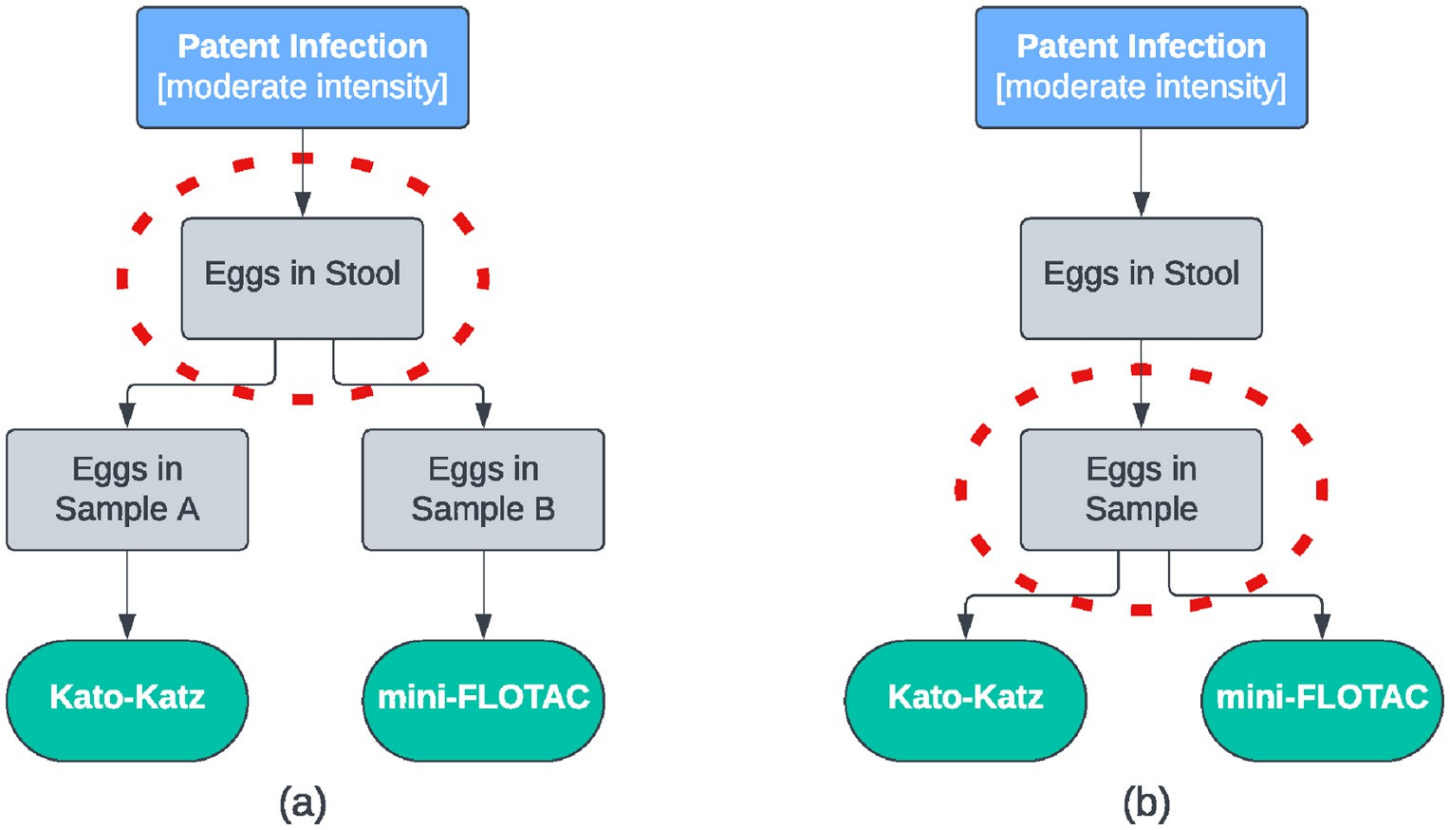
The target condition (blue), unobserved states (grey), test results (green), and approximate case definition (red circle) for 2 egg counting methods based on separate stool samples (a) or the same stool sample (b).

**Fig 3:**
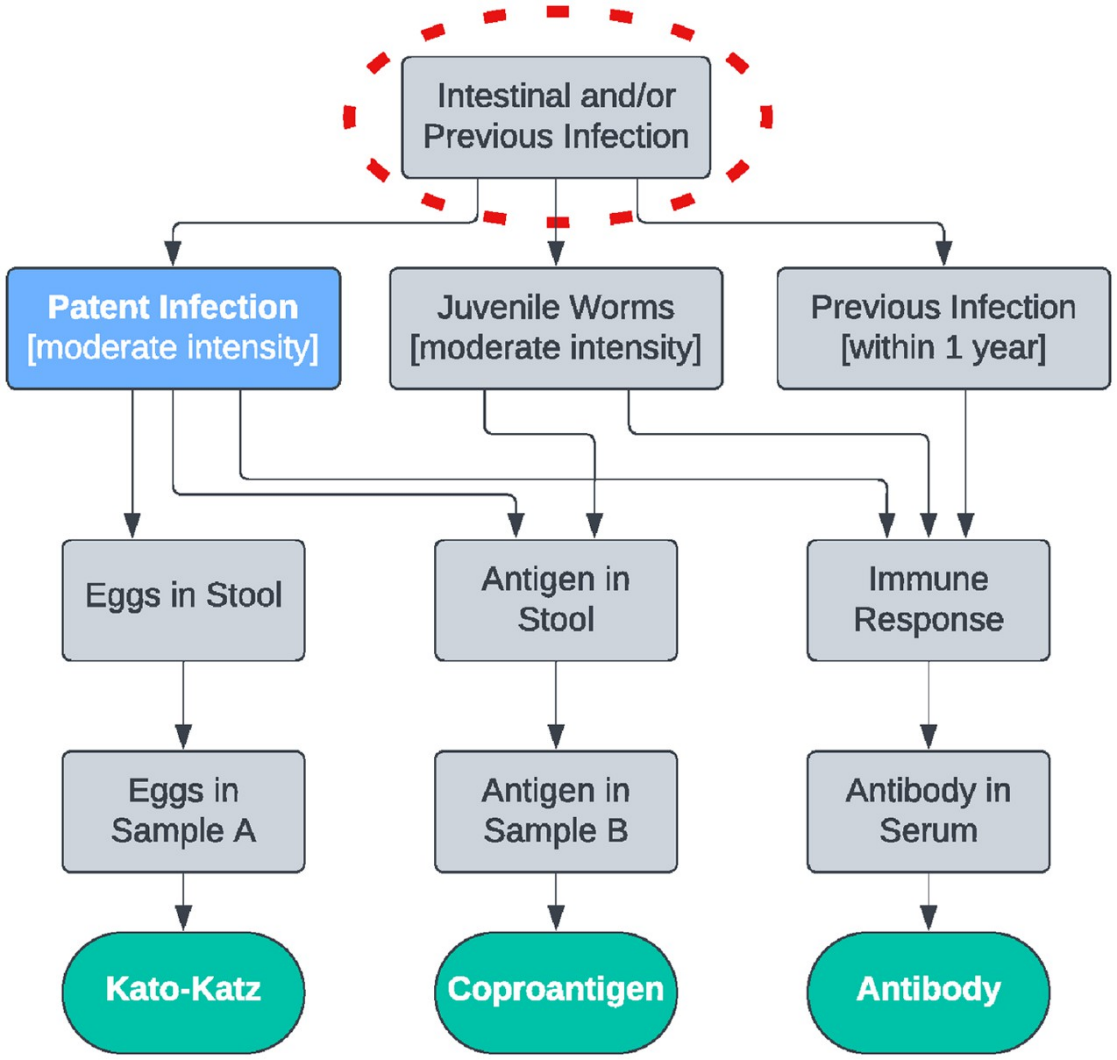
The target condition (blue), unobserved states (grey), test results (green), and approximate case definition (red circle) for Kato-Katz, coproantigen, and antibody tests.

There are 3 key outcomes of this visualisation process. The first is that the “highest common ancestor” of the DAG approximates the case definition implicitly defined by the choice of tests. Our stated target condition is “Patent Infection,” but because half of the tests detect both juvenile and adult worms, the case definition is closer to “Intestinal Infection.” This can be contrasted with the use of only Kato-Katz and mini-FLOTAC tests (Fig 2), where the implicit case definition is either “Eggs in Stool” or “Eggs in Sample,” depending on whether the tests are run on different stool samples from the same child (Fig 2a) or the same stool sample (Fig 2b). This inconsistency in the case definition may lead to different estimates of sensitivity and specificity across the 3 examples. We argue that the target condition is close to the case definitions in Figs 1 and 2a because the presence of parasite eggs is synonymous with patent infection, although pre-patent and/or single-sex infections will cause “Intestinal Infection” to differ from “Patent Infection.” The case definition in Fig 2b is quite different, so we would expect the sensitivity of both tests to be substantially higher than for the scenario in Fig 2a. In Fig 3, the inclusion of an antibody test alters the case definition to include previous infection, thus decreasing the sensitivity and specificity of the Kato-Katz and coproantigen tests. In this situation, it is necessary to exclude the antibody test to bring the case definition closer to the target condition.

The second key outcome is identifying pairs (or groups) of tests with common branches at an intermediate stage between the case definition and the test results, which would indicate a lack of conditional independence. In our case (Fig 1), “Eggs in Stool” is common to PCR, Kato-Katz, and mini-FLOTAC tests, while “Juvenile Worms” is common to PCR and coproantigen tests. It is important to account for this when analysing the data; this may be achievable using an appropriate LCM [7–10], but it may be beneficial to exclude data from one or more tests to reduce the complexity of the analysis.

The third key outcome is to identify any variation among populations in terms of interpretating the states and/or strength of connection between states. For Fig 1, the definition of “Patent Infection” as typical to an endemic setting, i.e., moderate/high burden, as well as the relative strength of connections between “Intestinal Infection” and “Patent Infection” versus “Juvenile Worms,” should be consistent among populations used in the study and populations in which the tests will be used in the future. For example, including a population with typically lower-intensity adult infection and/or a population with a substantially higher prevalence of juvenile-only infections relative to patent infections would change the case definition and therefore reduce the sensitivity of egg detection methods. This is typically difficult to verify purely on biological grounds, so should also be assessed during data analysis.

## Discussion

With traditional approaches to diagnostic test evaluation, the case definition is implicitly defined as the result of the reference standard. For example, if the reference test is based on clinical signs, then the case definition is “overt symptoms.” For LCM, the case definition (frequently referred to as the “latent class,” emphasising its hidden nature) is implicitly defined by the combination of diagnostic tests used. In either case, the case definition must be carefully considered by the user and reported as part of the results [19,24]. A graphical illustration (e.g., DAG) helps to determine the case definition by explicitly considering the biology of the system and target mechanisms of the diagnostic tests used. This approach may be unnecessary for trivial applications where the latent class is obvious, e.g., where all tests are designed to detect independent IgG antibodies so that the case definition is “IgG response” rather than “antibody response.” However, the standardised framework presented here is generally useful for helping readers unfamiliar with the biology to interpret the results. The framework also makes nuanced details of sampling/processing methods explicit, e.g., where multiple egg detection methods are used on the same faecal sample so that the latent class is closer to “presence of eggs in the sample” than to “patent infection.”

Where possible, the framework should be considered before data are collected, so that there is an opportunity to modify the combination of tests and/or populations used. In these cases, the DAG can be drawn starting at the desired target condition and “working down” to determine the profile of diagnostic tests that will give a case definition close to this target condition. However, we anticipate that the framework will also be used in situations where it is not possible to change the tests or populations used, either because no suitable alternative tests exist or because the data have already been collected. In these cases, the post hoc use of the framework serves to identify any discrepancies between the desired target condition and the implicit case definition by drawing the DAG starting at the tests used and “working up” to determine the implicit case definition. For our *A*. *lumbricoides* example, Fig 2b illustrates a problematic scenario because the case definition of “Eggs in Sample” is much narrower than the desired target condition of “Patent Infection.” In this situation, the framework can either be used prospectively to explore modification of the sampling protocol, or retrospectively to illustrate the major limitation of the study and associated caveat when interpreting results. Conversely, Fig 3 shows a scenario where the case definition is substantially broader (and more complex) than “Patent Infection,” illustrating the fact that including additional tests is not always advantageous. In this situation, the framework can be used to justify excluding the antibody test from the analysis either retrospectively by discarding the collected data, or prospectively by redistributing the available resources to a different test or increased sample size. Similarly, where the framework highlights potential discrepancies in test performance among populations, this information can be used to justify excluding the non-similar population(s). The framework also highlights groups of tests that detect similar targets and are therefore not conditionally independent. We note that if 2 tests agree almost perfectly then including both tests provides no additional information over including either one of the tests alone. Depending on the goal of the study, it may therefore be wise to reconsider the selected test profile to avoid using highly related tests.

As stated previously, all estimates of sensitivity and specificity must be interpreted in the context of the target condition and population. This offers the flexibility to use different target conditions for different purposes, e.g., evaluation of multiple antibody tests will yield a case definition of previous infection, which is relevant for estimating seroprevalence. It may also be convenient to define the target condition as the presence of a specific analyte in controlled samples for the purposes of diagnostic test development within a laboratory setting. While this is acceptable, the estimated sensitivity and specificity cannot be extrapolated to other target conditions. However, it is possible to incorporate prior information when using LCM implemented within a Bayesian statistical framework, potentially including an assumption of near-perfect specificity for reference tests based on direct detection.

This brief overview provides a useful heuristic device that can be used to obtain insight into the intricacies of specifying and interpreting results from diagnostic test evaluation studies using either LCM or a presumed gold standard. However, further guidance is needed to establish a standardised framework for use with NTDs, building upon the STARD-BLCM reporting guidelines [19]. A best-practice document for diagnostic test evaluation in the context of NTDs has been produced [25], on which we would like to invite comment from the wider NTD community. This can be freely downloaded from https://www.costmodds.org/testeval/ntd/report/.

## References

[1] Hawkins DM, Garrett JA, Stephenson B. Some issues in resolution of diagnostic tests using an imperfect gold standard. Stat Med. 2001;20:1987–2001. doi: 10.1002/sim.819 11427955

[2] McKenna SLB, Dohoo IR. Using and interpreting diagnostic tests. Vet Clin North Am Food Anim Pract. 2006;22:195–205. doi: 10.1016/j.cvfa.2005.12.006 16517302

[3] Schiller I, Van Smeden M, Hadgu A, Libman M, Reitsma JB, Dendukuri N. Bias due to composite reference standards in diagnostic accuracy studies. Stat Med. 2016;35:1454–1470. doi: 10.1002/sim.6803 26555849

[4] Limmathurotsakul D, Turner EL, Wuthiekanun V, Thaipadungpanit J, Suputtamongkol Y, Chierakul W, et al. Fool’s Gold: Why imperfect reference tests are undermining the evaluation of novel diagnostics: A reevaluation of 5 diagnostic tests for leptospirosis. Clin Infect Dis. 2012;55:322–331. doi: 10.1093/cid/cis403 22523263 PMC3393707

[5] Wilks C. Gold standards and fool’s gold. Aust Veterinary J. 2001;79:115–115. doi: 10.1111/j.1751-0813.2001.tb10717.x 11256281

[6] Hui SL, Walter SD. Estimating the error rates of diagnostic tests. Biometrics. 1980;36:167. doi: 10.2307/2530508 7370371

[7] Georgiadis MP, Johnson WO, Gardner IA, Singh R. Correlation-adjusted estimation of sensitivity and specificity of two diagnostic tests. J R StatSoc Series C: Appl Stat. 2003;52:63–76. doi: 10.1111/1467-9876.00389

[8] Liu Y, Ying G, Quinn GE, Zhou X, Chen Y. Extending Hui-Walter framework to correlated outcomes with application to diagnosis tests of an eye disease among premature infants. Stat Med. 2022;41:433–448. doi: 10.1002/sim.9269 34859902 PMC8884176

[9] Qu Y, Tan M, Kutner MH. Random Effects Models in Latent Class Analysis for Evaluating Accuracy of Diagnostic Tests. Biometrics. 1996;52:797. doi: 10.2307/2533043 8805757

[10] Wang Z, Dendukuri N, Zar HJ, Joseph L. Modeling conditional dependence among multiple diagnostic tests. Stat Med. 2017;36:4843–4859. doi: 10.1002/sim.7449 28875512

[11] Boelaert M, El Safi S, Goetghebeur E, Gomes-Pereira S, Le Ray D, Van der Stuyft P. Latent class analysis permits unbiased estimates of the validity of DAT for the diagnosis of visceral leishmaniasis. Trop Med Int Health. 1999;4:395–401. doi: 10.1046/j.1365-3156.1999.00421.x 10402977

[12] Tamarozzi F, Guevara ÁG, Anselmi M, Vicuña Y, Prandi R, Marquez M, et al. Accuracy, acceptability, and feasibility of diagnostic tests for the screening of Strongyloides stercoralis in the field (ESTRELLA): a cross-sectional study in Ecuador. Lancet Glob Health. 2023:1–9. doi: 10.1016/S2214-109X(23)00108-0 36972722

[13] Nikolay B, Brooker SJ, Pullan RL. Sensitivity of diagnostic tests for human soil-transmitted helminth infections: a meta-analysis in the absence of a true gold standard. Int J Parasitol. 2014;44:765–774. doi: 10.1016/j.ijpara.2014.05.009 24992655 PMC4186778

[14] Kazienga A, Levecke B, Leta GT, De Vlas SJ, Coffeng LE. A general framework to support cost-efficient survey design choices for the control of soil-transmitted helminths when deploying Kato-Katz thick smear. Cheng Q, editor. PLoS Negl Trop Dis. 2023;17:e0011160. doi: 10.1371/journal.pntd.0011160 37347783 PMC10321644

[15] Bärenbold O, Raso G, Coulibaly JT, N’Goran EK, Utzinger J, Vounatsou P. Estimating sensitivity of the Kato-Katz technique for the diagnosis of Schistosoma mansoni and hookworm in relation to infection intensity. French M, editor. PLoS Negl Trop Dis. 2017;11:e0005953. doi: 10.1371/journal.pntd.0005953 28976979 PMC5643140

[16] Cools P, Vlaminck J, Albonico M, Ame S, Ayana M, José Antonio BP, et al. Diagnostic performance of a single and duplicate Kato-Katz, Mini-FLOTAC, FECPAKG2 and qPCR for the detection and quantification of soil-transmitted helminths in three endemic countries. Freeman MC, editor. PLoS Negl Trop Dis. 2019;13:e0007446. doi: 10.1371/journal.pntd.0007446 31369558 PMC6675048

[17] Coffeng LE, Vlaminck J, Cools P, Denwood M, Albonico M, Ame SM, et al. A general framework to support cost-efficient fecal egg count methods and study design choices for large-scale STH deworming programs–monitoring of therapeutic drug efficacy as a case study. PLoS Negl Trop Dis. 2023;17:1–23. doi: 10.1371/journal.pntd.0011071 37196017 PMC10228800

[18] Greiner M, Gardner IA. Epidemiologic issues in the validation of veterinary diagnostic tests. Prev Vet Med. 2000;45:3–22. doi: 10.1016/s0167-5877(00)00114-8 10802331

[19] Kostoulas P, Nielsen SS, Branscum AJ, Johnson WO, Dendukuri N, Dhand NK, et al. STARD-BLCM: Standards for the reporting of diagnostic accuracy studies that use bayesian latent class models. Prev Vet Med. 2017;138:37–47. doi: 10.1016/j.prevetmed.2017.01.006 28237234

[20] Gardner IA, Colling A, Greiner M. Design, statistical analysis and reporting standards for test accuracy studies for infectious diseases in animals: Progress, challenges and recommendations. Prev Vet Med. 2019;162:46–55. doi: 10.1016/j.prevetmed.2018.10.023 30621898

[21] Knopp S, Mgeni AF, Khamis IS, Steinmann P, Stothard JR, Rollinson D, et al. Diagnosis of soil-transmitted helminths in the era of preventive chemotherapy: Effect of multiple stool sampling and use of different diagnostic techniques. Engels D, editor. PLoS Negl Trop Dis. 2008;2:e331. doi: 10.1371/journal.pntd.0000331 18982057 PMC2570799

[22] Coffeng LE, Graham M, Browning R, Kura K, Diggle PJ, Denwood M, et al. Improving the cost-efficiency of preventive chemotherapy: impact of new diagnostics on stopping decisions for control of schistosomiasis. Clin Infect Dis. 2024;78:S153–S159. doi: 10.1093/cid/ciae020 38662699 PMC11045014

[23] Digitale JC, Martin JN, Glymour MM. Tutorial on directed acyclic graphs. J Clin Epidemiol. 2022;142:264–267. doi: 10.1016/j.jclinepi.2021.08.001 34371103 PMC8821727

[24] Kostoulas P, Gardner IA, Elschner MC, Denwood MJ, Meletis L, Nielsen SS. Examples of proper reporting for evaluation (Stage 2 validation) of diagnostic tests for diseases listed by the World Organisation for Animal Health. Rev Sci Tech. 2021;40:287–298. doi: 10.20506/rst.40.1.3225 34140743

[25] Denwood M, Olsen A. Defining and Using Reference Standards for New Diagnostic Tests for Neglected Tropical Diseases. [cited 2024 Aug 9]. https://www.costmodds.org/testeval/ntd/report

